# Integrating Community Ecology into Models of Vector-Borne Virus Transmission

**DOI:** 10.3390/plants12122335

**Published:** 2023-06-15

**Authors:** Benjamin W. Lee, Liesl C. Oeller, David W. Crowder

**Affiliations:** 1 Department of Entomology and Nematology, University of California—Davis, Davis, CA 95616, USA; benjamin.w.lee@wsu.edu; 2 Department of Entomology, Washington State University, Pullman, WA 99163, USA; dcrowder@wsu.edu

**Keywords:** modeling, vector, virus, transmission, community ecology

## Abstract

Vector-borne plant viruses are a diverse and dynamic threat to agriculture with hundreds of economically damaging viruses and insect vector species. Mathematical models have greatly increased our understanding of how alterations of vector life history and host–vector–pathogen interactions can affect virus transmission. However, insect vectors also interact with species such as predators and competitors in food webs, and these interactions affect vector population size and behaviors in ways that mediate virus transmission. Studies assessing how species’ interactions affect vector-borne pathogen transmission are limited in both number and scale, hampering the development of models that appropriately capture community-level effects on virus prevalence. Here, we review vector traits and community factors that affect virus transmission, explore the existing models of vector-borne virus transmission and areas where the principles of community ecology could improve the models and management, and finally evaluate virus transmission in agricultural systems. We conclude that models have expanded our understanding of disease dynamics through simulations of transmission but are limited in their ability to reflect the complexity of ecological interactions in real systems. We also document a need for experiments in agroecosystems, where the high availability of historical and remote-sensing data could serve to validate and improve vector-borne virus transmission models.

## 1. Introduction

Insect-borne plant viruses are a major threat to agricultural productivity and to the stability and function of natural ecosystems [1,2]. In recent years, devastating epidemics of plant viruses have occurred globally as insect vectors establish in regions where hosts have limited tolerance, and it is estimated that up to 10% of global agricultural production is lost to disease [3,4]. In addition, agricultural intensification, climate change, and associated human-mediated land use changes have broadened the epidemiological interface where viruses are transmitted between cultivated and unmanaged plants, promoting outbreaks of known and previously unrecognized viruses in crop systems [5]. The development of strategies to prevent such damage to plants once outbreaks occur requires a precise understanding of the vector, virus, and host plant biology, life history, and behavior. However, studies that experimentally assess the interactions between vectors, viruses, and hosts remain limited in scale and focus on only a few pathosystems due to the logistical challenges of replicating more realistic field conditions [6].

Mathematical models are used to simulate biological and environmental processes that affect the transmission of vector-borne pathogens through plant communities with a goal of informing management decisions in a variety of agricultural ecosystems [7,8]. Most of the early attempts to model insect-borne plant virus transmission used relatively simple frameworks that only considered vector population dynamics and weather as factors that could predict pathogen outbreaks [9]. Though generally non-specific, these simple models were often able to capture long-term dynamic cycling of outbreaks, host die-offs, and recovery rates in forest ecosystems. The measurements of the specific virus transmission characteristics and vector traits, such as reproduction and movement, were later incorporated into the models to better replicate in-field vector and disease dynamics and assess how variation in a broader number of environmental factors could impact virus prevalence and transmission over time [10,11]. The model frameworks have since expanded to examine how the variation in specific vector traits can affect virus transmission rates. These models have indicated that minor shifts in vector behaviors, such as the vector’s preference for infected as compared to uninfected plants, have the potential to significantly alter the trajectory of virus outbreaks [12,13]. For example, when vectors of non-persistent viruses have higher fitness on uninfected compared to infected plants, they are induced to move after probing the infected plants, and virus transmission is promoted [12,13]. In contrast, when vectors of persistent viruses have higher fitness on infected plants, they are encouraged to remain on the hosts for enough time to complete feeding bouts and transmit pathogens [12,13]. Such models have also been useful in identifying the understudied vector characteristics and behaviors that may affect transmission rates in pathosystems.

Although there are many models of insect-borne pathogens, few capture the breadth of the interactions that affect vectors and pathogens. For example, within food webs, vectors interact with individuals of other species, including predators, mutualists, and competitors, which can affect the vectors in a variety of ways that cascade to affect pathogen transmission. Interactions with other species, for example, can alter vector abundance through changes in reproduction and mortality and also alter vector behaviors, such as rates of movement and host preferences [14,15,16]. For example, predators often reduce the fitness of insect vectors by consuming them, but predators often increase vector movement, which can accelerate the transmission of viruses. While the importance of such ecological interactions on pathogen transmission has generally been recognized for directly transmitted pathogens (i.e., pathogens that do not require a vector for transmission) [17,18], the models of vector-borne viruses rarely capture the effects of species interactions [19]. Yet, such models are difficult to develop as the effects of species interactions on vectors are difficult to predict *a priori* [20,21,22], and the lack of empirical studies investigating plant virus transmission in a community context has hampered the development of conceptual models.

In this review, we discuss how the interactions between vectors, predators, mutualists, and competitors are likely to affect the rates of vector-borne virus transmission. We also review the studies published from 2016 to 2021 that modeled how insect vectors affected plant virus transmission (*n* = 9). Studies were included if the models examined how variation in a vector trait (i.e., dispersal, host preference, etc.) influenced virus prevalence. We describe how the models identify important mechanisms through which vectors affect transmission rates but are parameterized almost exclusively from laboratory or greenhouse studies that may poorly reflect natural ecosystems. To fill in these important gaps in knowledge, we document the need for certain community ecology experiments in agricultural systems that could be designed to better test the model predictions and improve the applicability of the models to management decision-making. Our overall goal is to promote more proactive management of vector-borne pathogen outbreaks through a combination of realistic models and field work that measure key vector traits.

## 2. Vector Traits Affecting Virus Transmission

Hemipteran insects, such as aphids, leafhoppers, whiteflies, and psyllids, transmit the vast majority of identified plant viruses with their piercing–sucking mouthparts [23,24]. For these vector species, several specific traits are assumed to be the main determinants of virus spread and transmission rates [25,26]. Insect reproductive rates, for example, by increasing the abundance of potential vector individuals, are directly and positively linked to transmission rates for a variety of pathogens. In contrast, greater insect mortality is directly and negatively linked to transmission rates for most pathogens. The propensity of insects to disperse between hosts, both locally and across long distances, also contributes greatly to transmission rates, given that nearly all vector-borne viruses are entirely dependent on vectors for inter-host movement [27]. In fact, for several systems, alterations of vector movement have been shown to impact the rates of virus transmission more strongly than alterations of vector abundance [19]. However, movement is not always associated with an increase in pathogen transmission. If vector movement is so great that feeding duration is short, pathogen transmission rates can decrease compared to scenarios where the vectors spend less time moving and more time feeding [19].

After they disperse off a host plant, the vector preferences for infected or uninfected hosts can shape the number of feeding events where pathogen transmission is possible [13]. For example, vectors need to travel between an infected host and a healthy one for transmission to occur, which is more likely if the vectors prefer infected over healthy plants. Individual feeding behaviors, such as the number of probes before sustained feeding or the duration of salivation and feeding phases, also determine the vectors’ ability to acquire and transmit pathogens (i.e., their transmission efficiency) [28,29]. Importantly, insect life history and the interactions between these traits can all affect transmission outcomes. For example, rapidly reproducing aphid colonies may quickly reach the carrying capacity on hosts and increase their host-to-host movement, generating a considerable number of dispersing, infectious vectors [30]. Alternatively, vectors that disperse frequently may encounter many susceptible hosts but not feed for long enough periods to acquire or transmit persistent viruses, generating large populations without similarly large increased transmission rates (or even declines in transmission) [27]. The transmission dynamics of vector-borne pathogens are, therefore, a complex function of individual vector traits, vector population characteristics, pathogen characteristics, and the interactions between these processes.

The contributions of specific vector traits to transmission rates can also vary with the transmission mode of viruses [27]. Non-persistently transmitted viruses (henceforth “non-persistent viruses”), which are acquired after brief feeding probes when virions can attach to host mouthparts but remain transmissible on vector mouthparts ephemerally, have been shown to benefit from the rapid dispersal of vectors from infectious hosts [19]. This means that the greater host-to-host movement generally increases transmission rates even if the feeding duration declines with the greater movement because the pathogens are acquired so rapidly during the feeding bouts. In contrast, persistently transmitted viruses (henceforth “persistent viruses”), which require prolonged feeding and circulation into aphid salivary glands [31], benefit from longer feeding bouts and increased vector reproduction [6]. For these viruses, a greater host-to-host movement may actually slow the rates of virus transmission if it results in a concurrent decrease in the time per feeding bout [19]. In turn, there is accumulating evidence that suggests viruses can enhance their transmission by altering vector behavior and physiology in ways that maximize the spread for a particular virus type and transmission mode [32,33,34]. For example, the persistent *Potato leafroll virus* alters plant chemistry to attract aphids and prolong feeding bouts, enhancing transmission [35,36]. Alternatively, the non-persistent *Cucumber mosaic virus* lowers host quality to induce rapid dispersal after feeding, increasing transmission at the cost of vector fitness [37]. The relative consistency of these modifications shows the importance of even minor shifts in vector behavior to the rates of virus transmission in the field. Indeed, across many families of viruses and insects, the impacts of the pathogen transmission mode on insect behavior and fitness appear to be fairly consistent and general [6].

## 3. Community Factors Affecting Virus Transmission

While vector traits determine transmission potential and the rates of pathogen spread through a host population, the vectors’ interactions with other species within food webs can generate variation in behavior and physiology that also affects virus transmission. However, the effects of altered food webs on disease risk can be difficult to predict, given the complex and variable nature of species interactions [38]. As individual vector behaviors can disproportionately affect pathogen transmission, the role of ecological interactions on virus transmission may not be captured by macro-level responses such as vector population size. Here we describe several types of species interactions and document cases where unpredictable outcomes for disease transmission occur.

Insect vectors are threatened by diverse guilds of predators that persistently capture and kill the vectors. Accordingly, predator effects on virus transmission have been viewed primarily through their effects on vector population size. For example, early studies assessing the predator’s impacts on vectors in crop fields show that predators can reduce virus spread by lowering the vector population size, especially during periods of high movement when virus transmission potential is highest [39]. The early models of vector-borne pathogen transmission that included predation accordingly examined the effects of predators on vector abundance exclusively while ignoring any possible effects on vector behavior [40]. However, the importance of non-consumptive effects has been increasingly recognized in affecting prey physiology and behavior, and this framework has only started being applied to vector-borne pathogens [41,42]. In response to predation, vectors engage in avoidance behaviors [43,44,45] that can influence their feeding, dispersal, and reproductive output and, in turn, virus transmission. For example, attack by natural enemies induced the green peach aphid *Myzus persicae* to increase host-to-host movement between pepper plants in a greenhouse, increasing transmission of the *Broad bean wilt virus 1* [46]. Alternatively, the leafhopper *Psammotettix alienus* will delay initial feeding and reduce the time spent ingesting phloem in response to predator risk, limiting its ability to transmit *Wheat dwarf virus* [47,48].

Though predator effects on vector populations and behavior are well known [49], their effects on virus transmission in natural systems have not been well explored. Finke [50] adapted an existing model of pathogen transmission from Moore et al. [40] to outline several non-consumptive mechanisms through which predators could influence vector-borne pathogen transmission. They found that the interactions between consumptive and non-consumptive pathways likely mediate the consequences of predation for disease risk. As predator attack rates are often directly dependent on prey density (functional response, [51]), continuous feedback between predator effects on prey abundance and behavior make predicting the net effects on transmission difficult. Only one known study has simultaneously evaluated the effect of predators on vector abundance, behaviors, and transmission outcomes in the field. Lady beetle predators were found to accelerate the transmission of *Pea enation mosaic virus* by displacing vectors to new hosts or to regions of hosts susceptible to inoculation despite overall reductions in vector abundance [52]. While such small-scale field experiments can provide evidence for possible non-consumptive mechanisms of predator effects, larger scale evaluations of predator effects are required to allow for realistic population dynamics to emerge. However, large manipulations of vectors, pathogens, predators, and host populations have proven logistically challenging for most vector-borne pathosystems in the field, limiting the ability to conduct experiments. Despite these difficulties, such experiments will be crucial to understanding and preventing virus outbreaks as climate change and land use changes drive range shifts and result in novel interactions between species and pathogens.

Non-vector herbivores and mutualists can also indirectly impact virus transmission through competition (or facilitation) with vectors and indirectly by altering host traits. Herbivory by competing species, for example, can affect when, where, and how vectors feed on hosts through displacement or by affecting host quality [53]. A study examining the interactions between a non-vector herbivorous weevil *Sitona lineatus* and the aphid vector *Acyrthosiphon pisum* showed that, on a shared host, the weevils accelerated the transmission of *Pea enation mosaic virus* by displacing the vectors to new hosts or to regions of hosts susceptible to inoculation [54]. Further work in this system showed that the weevils decreased the number of aphid vectors required to successfully inoculate hosts [55]. While not often included in the models, intra-specific competition could also affect vector feeding rates, reproductive output, and transmission rates through density-dependent processes. However, similar to studies surrounding the indirect effects of predation, limited data exist concerning how non-vector competitors affect transmission in natural systems.

## 4. Modeling Vector-Borne Virus Transmission

At their core, all models of vector-borne virus transmission incorporate vector–host contact rates and transmission efficiency to predict virus spread within the host and vector populations [10]. The models can then be expanded to evaluate how changes to specific traits of vectors, hosts, and environments can affect the rates of spread. However, the models vary in the type of framework used to project virus spread with some using state-variables (SIR models for example), while other models are based on differential equations [19,50]. Recent models of virus transmission have also expanded to explore how variation in vector traits, driven by an improved understanding of virus–vector or virus–host interactions, could affect transmission in natural systems (Table 1).

By exploring the contributions of individual vector traits to the transmission of both non-persistent and persistent viruses (and both propagating and non-propagating viruses), the models of virus spread have identified key concepts for understanding disease dynamics. For example, in models of *Barley yellow dwarf virus* (persistent) and *Potato virus Y* (non-persistent) transmission, Shaw et al. [13] found that the vector growth rate was the strongest contributor to the transmission of both viruses by driving the rates of departure from infected hosts. They also showed the importance of the vector’s preference for hosts of the opposite infectious status (e.g., infectious vector selecting uninfectious hosts) as a primary driver of transmission; in other words, viruses spread fastest when the vector’s host preferences differed from the type of host they were on. Donnelly et al. [72] also showed that changes to host attractiveness resulting from non-persistent virus infection can enhance the initial transmission by accelerating interplant movement but inhibit long-term spread by disincentivizing reproduction and lowering overall vector abundance. These models of virus dynamics point to the considerable interplay between vector abundance, behavior, and the interactions with hosts that can unpredictably alter the rates of virus spread. However, the experiments that isolate the effects of interactions on fitness are often confounded by alterations of movement, which has made it difficult to properly parameterize most models of vector-borne virus transmission with realistic trait values.

To build on the models that only include vectors and plant hosts, some models have begun exploring how multiple species could affect virus transmission. For example, Galimberti et al. [80] evaluated multiple scenarios of *Potato virus Y* transmission with communities of colonizing *Myzus persicae* and 20 non-colonizing aphid species having varying transmission efficiencies [76]. They found that non-colonizing aphids drove *Potato virus Y* transmission more than colonizing aphids, supporting the field results where *Potato virus Y* outbreaks have occurred in the absence of aphids [84]. Non-colonizing aphids were particularly important early in the growing season, and the authors recommended that control strategies need to focus on vector management before plant symptoms are apparent to prevent outbreaks [80]. Crowder et al. [19] similarly presented an inclusive model of species interactions between vectors, competitors, predators, and mutualists within food webs. In this study, they examined persistent and non-persistent virus transmission with varied vector reproduction, mortality, feeding behavior, and dispersal based on predicted effects of predation, competition, and mutualism. By altering only the vector traits due to interactions, the authors were able to create a multi-species model, even though only the vector abundance was tracked in the simulations (i.e., effects of the interacting partners were held constant regardless of vector abundance or infection status). For both virus types, the effects of species interactions on vector movement behaviors contributed more to transmission than the effects on fitness (but refer to Sisterson [12]). Though non-specific in the organisms involved, this model provides an intuitive framework for examining future interactions between vectors and non-vector species.

However, empirical studies documenting virus–vector and virus–host interactions often occur in small laboratory or greenhouse settings, and few include species other than the vector, pathogen, and host of interest. The limited scope of such studies used to parameterize the models of vector-borne virus transmission can limit their applicability to field scenarios. Of the twenty-six studies used to parameterize vector traits in nine recently published models, only six (23.1%) were conducted outside of a greenhouse or laboratory (Table 1). Given the role of environmental conditions, such as temperature and other abiotic stressors, on mediating transmission rates [85], extrapolating data from highly controlled scenarios to larger scales can ignore important variability in vector responses to changing conditions. Additionally, the data used by the models can often be unrepresentative of the actual transmission scenarios. For example, in the model of *M. persicae* transmission efficiency from Galimberti et al. [80], the movement parameters for *M. persicae* were based solely on estimates for initial flight of winged aphids rather than long-term movement [81]. Winged aphid morphs or ‘alatae’ are produced only infrequently during the year due to variation in intra-specific competition or predation [86] with a majority of in-field transmission thought to occur via wingless (‘apterous’) aphids moving from plant to plant [87,88]. While discrepancies such as these do not nullify the conclusions of such models, they do reduce the ability to prescribe management strategies for improved control of pathogen transmission based on realistic assumptions from field studies.

## 5. Evaluating Virus Transmission in Agricultural Systems

The frameworks for understanding vector-borne virus transmission that the models have generated suggest a need for further empirical testing. Through generalizations of vector traits and simulated disease dynamics, the models have identified scenarios where the interactions between vectors, hosts, pathogens, and other species could generate unpredictable outcomes for virus transmission. Importantly, the model analyses have unequivocally advocated for more field-based estimates of parameters and examinations of transmission to validate their predictions [13,19]. The difficulties in controlling for traits of vector and host species, pathogen strains, and surrounding communities have limited the ability for data from existing field studies to provide useful parameters [89]. To address this, we suggest controlled studies in agricultural systems to expand the scope and relevancy of the available data. Ideally, studies should be designed to try and isolate the effects of various interactions on vector fitness, behavior, and transmission efficiency (these traits are often inter-related and difficult to tease apart without careful designs). One method for doing this successfully is to use structural equation modeling, where the effects of specific interactions on vector behavior and fitness can be isolated [54,55].

Agricultural fields have several characteristics that indicate their usefulness in supporting the models of vector-borne virus transmission. A massive amount of data is available that documents the traits and phenology of the prevalent pest and natural enemy communities through sampling and empirical studies [90], growth and resistance traits of host plants through a variety of research [91,92], and historical and remote-sensing detection of pest disease risk in growing regions [1,93]. In the design of experiments, cropping systems can also be selected that have known characteristics suitable for examining the model predictions, such as uniform disease tolerance in host populations, the presence of specific vectors, or the presence of specific community members that may affect the vectors (i.e., competitors, predators, mutualists). The relative biological simplicity of agricultural compared to natural systems can also benefit experimentation involving community interactions and virus transmission. Crop fields generally consist of genetically uniform, evenly spatially distributed host populations with reduced arthropod biodiversity [90,94]. In such scenarios, vectors interact with relatively few predator and competitor species as compared to natural ecosystems, allowing for the observed community effects to be attributed to the interactions with specific species. For example, in the Palouse region of eastern Washington and northern Idaho in the Northwestern United States, dry pea production is threatened primarily by two species of insect herbivores, the aphid vector *Acyrthosiphon pisum* and the herbivorous weevil *Sitona lineata*, and the aphid-transmitted *Pea enation mosaic virus* [95]. While *A. pisum* vectors are attacked by a diverse guild of arthropod predators [96], the predatory lady beetle *Hippodamia convergens* is consistently numerically dominant in the Palouse region (Lee, personal observation). The limited number of drivers of vector behavior in this system has enabled studies to evaluate virus transmission within realistic food webs [52,55]. The food web models from these empirical studies have shown great utility in measuring the key vector traits that drive the pathogen transmission rates through both direct and indirect pathways [54,55]. The large datasets of climate and imaging data from remote sensing technologies may also be useful in predicting where important vectors, hosts, and non-vector species could be co-occurring [97], allowing for a broader applicability of the results from empirical studies.

Some models of pathogen transmission can be used in agricultural decision-support tools, allowing growers to make more cost-efficient and effective management decisions. Examples of commonly used pathogen models are fire blight [98] and apple scab [99] in tree fruit systems. These fungal pathogens are easier to model because they are not vectored by insects; they can be usefully modeled based on environmental conditions, such as air temperature, humidity, precipitation, and wind. However, the vector-borne virus transmission models are more difficult to put into practice in agricultural systems. These models have been used to drive theory and improve understanding of the factors that impact virus spread rather than used to implement in-field management decisions to reduce the rate of infection spread. In an ideal world, growers would use the vector-borne virus models to predict the rate of infection spread and make management decisions based on the perceived risk to their entire crop. In reality, once pathogens are found in a field or orchard, growers will act immediately to try to cut off the spread completely (Oeller, personal communication). In addition, adding vector–pathogen dynamics into a model designed for agriculture is highly complex due to the huge number of interactions at play. This has led some U.S.-based decision-support tools, such as the Washington State University Decision Aid System [100], to use vector population models as a proxy for pathogen models. As vector populations increase, there is a higher risk for potential pathogen spread, and management timing is based on that premise. Another potential method for modeling vector-borne pathogens is to combine vector population models with vector trapping. Testing vectors for the target pathogen can help estimate the level of infected vectors in a location. Growers can set management thresholds for vector populations that reach a certain level and lower those thresholds for infected vectors. While there are still knowledge gaps in applying the vector-borne virus models to agricultural systems, many decision-support tools across the country are working to provide growers with models to aid in making effective management decisions.

## 6. Goals for Future Studies

It is important to recognize that the efforts to examine virus transmission in agroecosystems have been understandingly limited given the difficulties in conducting field experiments using pathogens. Finding experimental sites where viruses can be introduced and isolated is difficult, and some vectors such as leafhoppers disperse across habitats too broad to be manipulated [101], which can limit the study systems available for investigation. Additionally, the variable spatial and temporal dynamics of plant pathogens and the lack of reliable early detection associated with many pathogens make it difficult to identify the factors contributing to the initial introduction of viruses [102]. In the face of these limitations, we suggest that the primary objective for future studies should be to generate field-based estimates of vector life history, behavior, and transmission efficiency in representative ecological conditions. As much as possible, such studies should be conducted over realistic time frames (i.e., entire growing seasons or several years) rather than short time frames (i.e., days or weeks) to generate results that are applicable for commercial agriculture.

As previously described, the existing models of vector-borne virus transmission parameterized vector traits using life history data from lab or greenhouse studies almost exclusively (Table 1). Vector reproduction and development varies substantially between greenhouse and field studies with variable temperatures, habitat complexity, microclimates, and host plant characteristics in the field [103,104,105]. As vector abundance serves as the driver for vector behaviors in many models (e.g., vectors disperse once a host plant reaches carrying capacity [13]), system-specific estimates of abundance could greatly improve model predictions. The measurements of vector reproduction, development, and rates of interplant movement on the host species throughout the season would incorporate environmental variability important to making generalized predictions about emergent vector dynamics. Additionally, with these data, more models can exploit the detailed sensitivity analyses to show how the predictions of infectious disease spread might change under realistic values for vector fitness and behavior.

The field experiments in such systems could be expanded upon to examine further levels of complexity. For example, the field mesocosms used to evaluate vector traits could include predator or competitor species to evaluate the short-term effects of species interactions on vector responses, as in Lee et al. [52]. Alternatively, studies could compare vector population changes between closed mesocosms and those open to other species and attempt to identify the causal relationships through community sampling. Experiments examining the direct and indirect effects of virus infection on vector traits are also required. Dozens of studies have demonstrated how pathogen infection can alter host chemical defense and nutritional quality in ways that influence individual vector reproduction and behavior, though these effects have not been well documented in the field [33,34]. Further work measuring vector population dynamics across varied levels of virus prevalence in host populations could provide a better understanding of the long-term implications of virus-altered host phenotypes. Using these relatively simple and straightforward experimental approaches to generate data in pathosystems of interest would greatly reduce the gap between theoretical and applied models of transmission.

## 7. Conclusions

Predicting vector-borne virus transmission in natural and agricultural systems requires an abundance of data on vectors, viruses, hosts, and community interactions. Models have expanded our understanding of disease dynamics through simulations of transmission based on vector traits and their assumed relationships with other factors, but most models are limited in their ability to reflect the complexity of ecological interactions in real systems. Further experimentation using representative communities and environmental conditions are needed to examine how vectors respond to realistic scenarios and improve future models of transmission. We suggest that future studies in agricultural systems could provide the controlled conditions needed to parameterize models while providing opportunities for the emergent effects of species interactions to manifest.

## Figures and Tables

**Table 1:** Recent (2016–2021) modeling studies examining how vector-trait variation affects plant virus transmission.

Traits Examined	Virus	Vector spp.	Parameters Derived from?	Reference
Vector Reproduction, Mortality, Dispersal, Feeding Duration	Cucumber Mosaic Virus (CMV)	*Aphis gossypii*	Greenhouse: [56,57]	[19]
	Barley Yellow Dwarf Virus (BYDV)	*Rhopalosiphum padi*	Field: [58], Greenhouse: [59,60,61]	
Vector Reproduction, Host preference, Dispersal, Infectious status	Potato Virus Y (PVY)	*Myzus persicae*	Greenhouse: [32,62,63]	[13,64]
	Barley Yellow Dwarf Virus (BYDV)	*R. padi*	Greenhouse: [65], Field: [66]	
Vector Reproduction, Mortality	Non-persistent vs Persistent	*Non-specific*	Model: (based on [11], parameterized using [23] review)	[67]
Vector Reproduction, Incubation Period,	Potato Virus Y (PVY)	*M. persicae*	Greenhouse: [62,68,69]	[70]
	Barley Yellow Dwarf Virus (BYDV)	*R. padi*	Greenhouse: [71]	
Vector Host Preference, Dispersal, Feeding Duration,	Non-persistent Virus	*Non-specific*	None stated	[72]
Vector Transmission Efficiency, Aggregation	Cassava Mosaic Virus	*Bemesia tabaci*	Field: [73,74]	[75]
Vector Abundance, Dispersal, Transmission Efficiency, Initial Inoculum,	Potato Virus Y (PVY)	*Multiple aphid species*	Greenhouse: [76,77,78,79]	[80]
			Field: [81,82]	
Host Plant Nutritional Status, Vector Transmission Efficiency	Barley Yellow Dwarf Virus (BYDV) and Cereal Yellow Dwarf Virus (CYDV)	*R. padi*	Greenhouse: Measured in this studyModel: [18], unparameterized conceptual model	[83]

## Data Availability

No new data were created or analyzed in this study. Data sharing is not applicable to this article.
